# Modeling injury severity among motor vehicle occupants using a safe system–aligned, population-based framework: evidence from Ohio crash data (2017–2023)

**DOI:** 10.1186/s40621-026-00676-3

**Published:** 2026-04-06

**Authors:** Angela L. Harden, Mary E. Cole, Brian Bautsch, Brittany Shoots-Reinhard, Christopher Kinn, Lauren Cardoni, Jeremy Thompson, John H. Bolte

**Affiliations:** 1 School of Health and Rehabilitation Sciences, Injury Biomechanics Research Center, College of Medicine, The Ohio State University Wexner Medical Center, 333 W 10th Avenue, 2063 Graves Hall, Columbus, OH 43210 USA; 2 School of Health and Rehabilitation Sciences, Division of Radiologic Sciences and Therapy and Injury Biomechanics Research Center, College of Medicine, The Ohio State University Wexner Medical Center, 453 W 10th Avenue, Columbus, OH 43210 USA; 3 American Honda Motor Co., Inc, 1919 Torrance Boulevard, Torrance, CA 90501 USA; 4 College of Arts and Sciences, Department of Psychology, The Ohio State University, 1835 Neil Avenue, Columbus, OH 43210 USA; 5 Department of Public Safety, Ohio State Highway Patrol, 1970 W Broad Street, Columbus, Ohio, OH 43223 USA; 6 Burgess & Niple, 330 Rush Alley Suite 700, Columbus, OH 43215 USA; 7 Highway Safety Program, Department of Transportation, 1980 W Broad Street, Columbus, Ohio, OH 43223 USA; 8 Injury Biomechanics Global, Inc, 6095 Whittingham Dr, Dublin, OH 43017 USA

**Keywords:** Crash injury prediction, Risk modeling, Traffic safety analysis, Multivariable risk assessment, Systemic crash factors, Vision Zero

## Abstract

**Background:**

Motor vehicle crashes remain a leading cause of serious injury and death in the United States. Although individual crash risk factors are well documented, less is known about how multiple risk factors co-occur across system domains to influence injury severity. This study applies a Safe System framework to estimate the population-level probability of suspected serious injury (SSI) or fatality associated with combinations of crash-related factors spanning the domains of People, Vehicle, Road, and Speed using statewide Ohio crash data from 2017 to 2023.

**Methods:**

Crash records from the Ohio Department of Public Safety were analyzed using multivariable generalized linear models. Person-, vehicle-, and crash-level variables were classified into four Safe System domains. Regression models were used to estimate adjusted odds and predicted probabilities of SSI or fatality associated with individual risk factors and combinations of co-occurring factors. Marginal effects were calculated to quantify changes in predicted risk across varying risk profiles.

**Results:**

Behavioral factors, including driver impairment and lack of restraint use, were associated with the largest increases in predicted probability of serious or fatal injury. Vehicle factors, such as older model year, and roadway characteristics, including roadway departure and curved alignments, also contributed significantly to injury risk. The highest predicted probabilities of severe outcomes occurred when multiple risk factors were present simultaneously across Safe System domains, demonstrating the compounding nature of injury risk within real-world crash environments.

**Conclusions:**

Serious and fatal crash outcomes are driven by interactions among risk factors across People, Vehicle, Road, and Speed domains. Consistent with Safe System principles, the most severe outcomes arise when layers of protection fail simultaneously. By quantifying conditional probabilities for specific combinations of risk factors, this study provides a practical, scalable, and data-driven framework for identifying system vulnerabilities and prioritizing integrated countermeasures. Strategies that simultaneously address behavioral risks, modernize the vehicle fleet, improve roadway design, and manage travel speeds are likely to produce greater reductions in SSI and fatalities than single-domain approaches. These findings support a shift toward coordinated, multi-domain safety programs and equitable vehicle and infrastructure investments as the foundation for future transportation safety and Vision Zero efforts.

## Background

Traffic crashes remain a persistent public health burden, shaped by interacting factors across road users, vehicles, roadway environments, and speed conditions. Although behaviors such as distraction [1, 2], impairment [3–5], fatigue [6], and speeding [7, 8] are frequently documented in crashes resulting in suspected serious injuries (SSI) and fatalities, these behaviors occur within broader system contexts that magnify the consequences of human error. Older vehicles lacking modern crash protection, high-speed corridors, adverse weather, and roadway designs with minimal tolerance for mistakes all contribute to environments where relatively small errors can result in severe harm. These factors rarely operate independently; the convergence of multiple vulnerabilities increases the likelihood that injuries will be serious or fatal.

The Safe System Approach (SSA) [9–11] addresses this complexity by recognizing that human mistakes are inevitable, but serious injuries and fatalities are not. The SSA emphasizes shared responsibility across transportation system designers, policy makers, and road users and focuses on building system elements that anticipate and absorb human error. Despite this emphasis on interactions across behavioral, vehicular, roadway, and speed-related domains, empirical crash research has typically examined these domains separately, producing a fragmented understanding of how injury severity emerges from their combined effects. Traditional safety programs and countermeasure frameworks often categorize risks independently, which obscures how moderate individual risks, such as restraint nonuse, older vehicle age, or curve negotiation, can become high-risk when co-occurring.

Despite growing adoption of the Safe System Approach in transportation safety policy, empirical analyses that operationalize this framework remain limited [12–15]. A large body of crash severity research has traditionally relied on statistical models that evaluate individual risk factors, such as driver behavior, roadway characteristics, or environmental conditions, independently when examining injury outcomes [16–18]. These studies commonly use ordered injury severity models or similar regression-based approaches that treat injury outcomes along a single ordinal scale rather than explicitly examining the transition to suspected serious injury or fatal outcomes [19, 20]. While such approaches have generated valuable insights into crash risk, they often do not explicitly evaluate how conditions across Safe System domains, People, Vehicles, Roads, and Speed, interact to shape the likelihood of severe harm. Recent reviews of Safe System research emphasize that although the framework promotes system-wide safety thinking, empirical studies that integrate multiple Safe System pillars within a unified analytical structure remain relatively limited in the literature. As transportation agencies increasingly adopt Safe System principles for strategic safety planning, there is a need for analytical approaches that move beyond siloed factor analysis and instead evaluate the combined influence of system elements on the probability of high-severity outcomes.

A Safe System–aligned analytical approach must therefore evaluate injury severity within the full system context rather than isolating contributing factors. To support integrated safety planning [21], the objective of this study is to quantify the conditional probability of suspected serious injury and fatality given specific combinations of People-, Vehicle-, Road-, and Speed-related factors. The goal of this research is not to determine causation or fault but to identify system-level patterns associated with disproportionately high harm.

Ohio’s statewide crash reporting system provides a comprehensive, multilevel dataset that captures detailed crash-, unit-, and person-level information across diverse transportation environments. Leveraging seven years of Ohio crash data (2017–2023), this study evaluates how co-occurring system conditions shape the severity of motor vehicle occupant injuries. By integrating Safe System domains into a unified modeling framework, the analysis provides a statewide probability-based assessment that moves beyond siloed risk evaluations and identifies combinations of system conditions associated with disproportionately high risks of suspected serious injury and fatality.

## Methods

### Data sources and case selection

All reported crashes on public roadways in Ohio are documented using the standardized Ohio Traffic Crash Report (OH-1) and submitted electronically by local and state law enforcement agencies to the Ohio Department of Public Safety (ODPS). The Ohio State Highway Patrol compiles, validates, and maintains these records through the Ohio Statistics and Analytics for Traffic Safety (OSTATS) system [22, 23]. Each OH-1 record includes linked information at three hierarchical levels, (a) crash-level circumstances (e.g., date, location, environmental conditions), (b) unit-level vehicle characteristics (e.g., body type, model year, contributing circumstances), and (c) person-level details (e.g., age, safety equipment use, injury severity).

Statewide crash data from 2017 to 2023, for all reported incidents, were obtained directly from the Ohio Department of Public Safety (ODPS). This period was selected to maximize statistical power while maintaining consistency in crash reporting definitions, variable availability, and coding practices across years. These years represent the most recent complete data available at the time of analysis and capture pre-pandemic, pandemic, and post-pandemic travel periods.

For this study, the person was the unit of analysis. All injured vehicle occupants meeting inclusion criteria were retained as individual observations, with their corresponding vehicle- and crash-level characteristics linked through the OH-1 hierarchical structure. Thus, the analytic dataset reflects person-level injury outcomes while incorporating contextual risk factors from the vehicle and crash levels.

Prior to analysis, all records underwent standardization and quality checks to ensure consistency across jurisdictions. Vehicle information was validated using the National Highway Traffic Safety Administration (NHTSA) VIN Decoder, and only vehicles with verifiable Vehicle Identification Numbers (VINs) were retained [24]. Duplicate records, incomplete vehicle entries, and non-resolvable inconsistencies were removed. Crashes involving pedestrians, bicyclists, or motorcyclists were excluded due to fundamentally different exposure patterns and injury mechanisms relative to enclosed-vehicle occupants.

Injury severity on the OH-1 is documented using a KABCO-aligned classification system, in which numeric codes correspond to fatal injury (1 = K), suspected serious injury (2 = A), suspected minor injury (3 = B), possible injury (4 = C), and no apparent injury (5 = O). For the present analysis, all vehicle occupants coded as sustaining possible injury (severity code 4), suspected minor injury (severity code 3), suspected serious injury (severity code 2) or fatal injury (severity code 1) were retained. Individuals with no reported injury severity were excluded from the analysis (*n* = 2,999,093) (Table 1). After cleaning and removal of unverifiable, non-applicable, or non-vehicle records, the final analytic dataset of persons with any injury (578,796) included 36,061 suspected serious injuries (SSIs) and 5,152 fatalities. This dataset al.so retained non-severe injury levels (possible injury, suspected minor injury) to calculate the predicted probability that any injury was an SSI or fatality. All data management and statistical analyses were performed in R version 4.3.2 [25].

**Table 1:** Frequency of Injury Severity by Crash Year

Crash Year	No Apparent Injury	Possible Injury	Suspected Minor Injury	Suspected Serious Injury	Fatal	Any Injury Total
**2017**	433,504	44,170	30,082	5,662	698	80,612
**2018**	495,461	49,864	33,415	5,595	695	89,569
**2019**	489,834	44,435	43,094	5,234	760	93,523
**2020**	379,275	36,200	37,903	4,847	766	79,716
**2021**	438,825	40,578	41,800	5,508	849	88,735
**2022**	335,766	29,132	31,064	4,043	615	64,854
**2023**	426,428	36,650	39,196	5,172	769	81,787
**2017–2023**	2,999,093	281,029	256,554	36,061	5,152	578,796

### Safe system domains and risk factor coding

Consistent with the Safe System Approach, all risk factors were categorized into four domains—People, Vehicle, Road, and Speed (Table 2). Each variable was derived by consolidating one or more standardized OH-1 fields. Every injured occupant was coded as either TRUE or FALSE for each risk factor based on the information linked to the corresponding person-, unit-, and crash-level records. When discrepancies occurred across fields (e.g., conflicting driver characteristics), the most specific and person-level field was prioritized, followed by unit-level and crash-level information.

People-domain factors captured unsafe or vulnerability-related characteristics of the driver or injured occupant. For example, Driver Impaired was coded as TRUE when the driver associated with the injured occupant’s vehicle had any documented indication of alcohol, drug, or medication impairment, including suspected involvement when confirmed toxicology was unavailable. Driver Distracted was coded as TRUE when the driver associated with the injured occupant’s vehicle had any distraction source listed in the “Driver Distracted By” sequence. No Belt Used was coded as TRUE for injured occupants aged nine years or older whose “Safety Equipment Used” field indicated no restraint. Age-related vulnerability was captured using OH-1 age group categories for injured occupants in vehicles with teen and senior drivers.

Vehicle-domain factors reflected characteristics of the vehicle in which the injured occupant was traveling. Vehicle Year was the only continuous variable and was converted to a binary indicator based on an empirical threshold. A series of binomial logistic regression models was estimated for each model year from 1981 to 2023 to examine the association between vehicle model year and the likelihood of an SSI or fatal outcome. In other words, separate models were used to estimate how the probability of an SSI or fatality changed across vehicle production years. Predicted marginal effects were then computed using the *marginaleffects* package [26], which translates model coefficients into adjusted changes in predicted probability, holding other variables constant. Across models, vehicles manufactured from 2011 to 2023 consistently showed lower predicted severity, whereas vehicles from 1981 to 2009 showed elevated severity. Model year 2010 fell at the inflection point and did not differ statistically from either group [27]. Based on this pattern, Vehicle Year < 2010 was coded as the elevated vehicle-risk category.

Road-domain factors represented roadway or maneuver circumstances preceding the crash. For example, Drove Off Road was coded as TRUE when the associated vehicle’s contributing circumstances included roadway departure. Negotiating a Curve was assigned as TRUE when the pre-crash action indicated curve navigation. Wrong-way travel was coded as TRUE using the corresponding contributing circumstance.

Speed-domain factors reflected unsafe or unstable speed-related behaviors documented by the reporting officer, including Unsafe Speed, Following Too Close, and Swerving to Avoid. These factors capture situations in which the vehicle’s travel speed or car-following behavior exceeded safe tolerance for roadway or traffic conditions.

**Table 2:** Safe system risk category and risk factor definitions

Safe system risk category	Risk factor	Risk factor definition
People	Driver Impaired	Injury to Person in Unit where Person Type “Driver” was: • “Condition” = “Under the Influence of Medications / Drugs / Alcohol” OR • “Is Alcohol Suspected” = “True” OR • “Is Marijuana Suspected” = “True” OR • “Is Other Drug Suspected” = “True”
	Driver Distracted	Injury to Person in Unit where Person Type “Driver” was “Driver Distracted By”: • “Manually operating an electronic communication device (texting, typing, dialing)” OR • “Talking on hands-free communication device” OR • “Talking on hand-held communication device” OR • “Other activity with an electronic device” OR • “Passenger” OR • “Other distraction inside the vehicle” OR • “Other distraction outside the vehicle”
	No Belt Used	Injury where Person was: • “Age” >= 9 AND • “Safety Equipment Used” = “None Used”
	Senior Driver	Injury to Person in Unit where Person Type “Driver” was: • “Age Group” = “Older Adult (61–79)” OR • “Age Group” = “Elderly Adult (80+)”
	Teen Driver	Injury to Person in Unit where Person Type “Driver” was: “Age Group” = “Teen (13–20)”
Vehicle	Vehicle < 2010	Injury to Person in Unit where: • “Vehicle Year” < 2010
Road	Negotiating a Curve	Injury to Person in Unit where: • “Pre-Crash Actions” = “Negotiating a Curve”
	Drove Off Road	Injury to Person in Unit where: • “Contributing Circumstances” = “Drove Off Road”
	Wrong Way	Injury to Person in Unit where: • “Contributing Circumstances” = “Wrong Way”
Speed	Unsafe Speed	Injury to Person in Unit where: • “Contributing Circumstances” = “Unsafe Speed”
	Following Too Close	Injury to Person in Unit where: • “Contributing Circumstances” = “Following Too Close”
	Swerving to Avoid	Injury to Person in Unit where: • “Contributing Circumstances” = “Swerving to Avoid”

### Frequency analyses

Relative frequencies were calculated to describe how often each Safe System category and individual risk factor appeared among suspected serious injuries and fatalities. Because a single injury event may involve several contributing factors, frequencies were estimated both for all cases associated with each factor and for the subset of cases in which only one factor or category was present. This allowed the analysis to distinguish between isolated conditions and situations in which multiple vulnerabilities co-occurred. Visualizations created using the *ggplot2* package provided a standardized graphical format for comparing the distribution of people-, vehicle-, road-, and speed-related conditions across injury outcomes [28]. These descriptive patterns offer a foundation for interpreting subsequent modeling results by illustrating how frequently specific system elements appear in the crash dataset.

### Co-occurrence analysis

Co-occurrence among Safe System factors was evaluated using UpSet visualizations generated with the *ComplexUpset* package [29]. This method provides a scalable solution for examining intersections among multiple variables and is particularly well suited for Safe System analysis, where overlapping conditions are expected rather than exceptional. The approach allows simultaneous assessment of how people-, vehicle-, road-, and speed-related factors combine within individual crashes, offering insight into the patterns of vulnerability that emerge when multiple system elements are involved.

For every observed combination of factors, the UpSet framework displays the number and percentage of suspected serious injuries or fatalities associated with that intersection, alongside the model-estimated probability that any injury with that combination of risk factors reaches the severity level of an SSI or fatality. Presenting observed frequencies together with predicted probabilities of injury severity allows the analysis to distinguish between conditions that occur often but tend to result in less severe injuries, conditions that are rare yet highly injurious, and conditions that are both frequent and associated with elevated severity. This integrated perspective highlights the system contexts in which risk becomes compounded and illustrates how injury severity reflects not only the presence of individual factors but the interaction of broader environmental, behavioral, vehicular, and speed-related conditions.

### Statistical modeling

Injury severity was modeled using proportional odds logistic regression, an approach suited to the ordered structure of the outcome, which included non-severe injury, suspected serious injury (SSI), and fatality. The proportional odds framework assumes that each predictor exerts a consistent directional effect across the thresholds separating the three severity levels. In practical terms, this means that a given risk factor (e.g., impairment or older vehicle model) is assumed to increase, or decrease, the odds of an occupant sustaining an SSI or resulting in a fatality when compared to the dataset of non-severe and severe crashes (excluding property damage cases). The model therefore estimates a single interpretable effect for each predictor or combination of predictors that reflects its overall association with an SSI or fatality. This approach allows for parsimonious modeling while preserving the ordered nature of the injury outcome and provides interpretable estimates of the likelihood that an injury falls into a more severe category under varying crash conditions. All Safe System factors explored in this study were included as predictors, and the model incorporated every observed combination of conditions that appeared in the dataset to ensure that results reflected real-world constellations of risk rather than hypothetical interactions.

The input dataset consisted of all injured person rows, with the person’s injury severity coded as either Non-Severe (e.g., Possible Injury or Suspected Minor Injury), Suspected Serious Injury, or Fatal. Injury severity was coded as an ordered factor, increasing from Non-Severe to Suspected Serious Injury to Fatal. Injured person rows were also coded as TRUE or FALSE for the presence of each risk factor and each risk category in association with that injury (described in Table 2). R package *MASS* [30] was used to fit an ordinal logistic regression model for binarized risk categories (Table 3) and a separate ordinal logistic regression model for binarized risk factors (Table 4).

**Table 3:** Model equations and model summary for risk categories

Non-Severe |SSI: logit (*P*(Y ≤ j)) = 3.25–0.73*People – 0.34*Vehicle – 0.77*Road – 0.13*Speed							
SSI|Fatal: logit (*P*(Y ≤ j)) = 5.41–0.73*People – 0.34*Vehicle – 0.77*Road – 0.13*Speed							
**Intercepts**	**Coefficient**	**Std. Error**	**t value**	**p-value**			
Non-Severe|SSI	3.25	0.01	330.97	*< 0.001*			
SSI|Fatal	5.41	0.02	329.68	*< 0.001*			
**Risk Category**	**Coefficient**	**Std. Error**	**t value**	**p-value**	**2.5% CI**	**97.5% CI**	**Odds Ratio**
People	0.73	0.01	68.56	*< 0.001*	0.71	0.75	2.08
Vehicle	0.34	0.01	32.92	*< 0.001*	0.32	0.36	1.41
Road	0.77	0.01	59.03	*< 0.001*	0.74	0.79	2.15
Speed	0.13	0.01	9.37	*< 0.001*	0.11	0.16	1.14

**Table 4:** Model equations and model summary for risk factors

Non-Severe |SSI: logit (*P*(Y ≤ j)) = 3.15–1.50*No Belt Used − 0.83*Driver Impaired – (−0.06)*Driver Distracted - (−0.08)*Teen Driver − 0.39*Senior Driver – 0.30*Vehicle < 2010 − 0.32*Negotiating a Curve − 1.43*Wrong Way − 0.44*Drove Off Road – 0.53*Unsafe Speed - (−0.27)*Following Too Close − 0.06*Swerving to Avoid							
SSI |Fatal: logit (*P*(Y ≤ j)) = 5.38–1.50*No Belt Used − 0.83*Driver Impaired – (−0.06)*Driver Distracted - (0.08)*Teen Driver − 0.39*Senior Driver – 0.30*Vehicle < 2010 − 0.32*Negotiating a Curve − 1.43*Wrong Way − 0.44*Drove Off Road – 0.53*Unsafe Speed - (−0.27)*Following Too Close − 0.06*Swerving to Avoid							
**Intercepts**	**Coefficient**	**Std. Error**	**t value**	**p-value**			
Non-Severe|SSI	3.15	0.01	336.64	*< 0.001*			
SSI|Fatal	5.38	0.02	329.31	*< 0.001*			
**Risk Factors**	**Coefficient**	**Std. Error**	**t value**	**p-value**	**2.5% CI**	**97.5%** **CI**	**Odds Ratio**
No Belt Used	1.50	0.01	114.02	*< 0.001*	1.48	1.53	4.49
Driver Impaired	0.83	0.02	51.08	*< 0.001*	0.80	0.86	2.29
Driver Distracted	−0.06	0.02	−2.51	*0.012*	−0.11	−0.01	0.94
Teen Driver	−0.08	0.02	−4.86	*< 0.001*	−0.11	−0.05	0.93
Senior Driver	0.39	0.01	27.90	*< 0.001*	0.37	0.42	1.48
Vehicle < 2010	0.30	0.01	27.78	*< 0.001*	0.28	0.32	1.35
Negotiating a Curve	0.32	0.02	15.70	*< 0.001*	0.28	0.36	1.37
Wrong Way	1.43	0.07	20.29	*< 0.001*	1.29	1.57	4.18
Drove Off Road	0.44	0.02	25.70	*< 0.001*	0.41	0.48	1.56
Unsafe Speed	0.53	0.02	24.98	*< 0.001*	0.49	0.57	1.70
Following Too Close	−0.27	0.02	−11.39	*< 0.001*	−0.31	−0.22	0.77
Swerving to Avoid	0.06	0.05	1.20	0.229	−0.04	0.15	1.06

Using the *predict* function in R package MASS [30], these models were used to estimate the probability of each injury severity outcome predicted for each TRUE/FALSE combination of risk categories (Table 5) or risk factors.

**Table 5:** Predicted Probabilities for All TRUE/FALSE Combinations of Risk Category

People	Vehicle	Road	Speed	Non-Severe Injury Probability (%)	SSI Probability (%)	Fatal Probability (%)
**FALSE**	**FALSE**	**FALSE**	**FALSE**	96.3%	3.3%	0.4%
**FALSE**	**FALSE**	**FALSE**	**TRUE**	95.7%	3.8%	0.5%
**FALSE**	**TRUE**	**FALSE**	**FALSE**	94.8%	4.6%	0.6%
**FALSE**	**TRUE**	**FALSE**	**TRUE**	94.1%	5.2%	0.7%
**TRUE**	**FALSE**	**FALSE**	**FALSE**	92.5%	6.6%	0.9%
**FALSE**	**FALSE**	**TRUE**	**FALSE**	92.3%	6.8%	1.0%
**TRUE**	**FALSE**	**FALSE**	**TRUE**	91.5%	7.4%	1.1%
**FALSE**	**FALSE**	**TRUE**	**TRUE**	91.3%	7.7%	1.1%
**TRUE**	**TRUE**	**FALSE**	**FALSE**	89.7%	9.0%	1.3%
**FALSE**	**TRUE**	**TRUE**	**FALSE**	89.4%	9.2%	1.3%
**TRUE**	**TRUE**	**FALSE**	**TRUE**	88.4%	10.1%	1.5%
**FALSE**	**TRUE**	**TRUE**	**TRUE**	88.1%	10.4%	1.5%
**TRUE**	**FALSE**	**TRUE**	**FALSE**	85.1%	12.9%	2.0%
**TRUE**	**FALSE**	**TRUE**	**TRUE**	83.4%	14.4%	2.2%
**TRUE**	**TRUE**	**TRUE**	**FALSE**	80.2%	17.0%	2.7%
**TRUE**	**TRUE**	**TRUE**	**TRUE**	78.0%	18.8%	3.1%

These predicted probabilities were then evaluated alongside the observed frequencies reported in the descriptive analyses to identify patterns that were common but relatively low-risk, rare but disproportionately severe, or both frequent and strongly injurious. This modeling strategy aligns with the Safe System emphasis on understanding how multiple system elements jointly influence the consequences of inevitable human error and highlights the contexts in which system vulnerabilities converge to produce elevated injury severity.

## Results

**Fig. 1:**
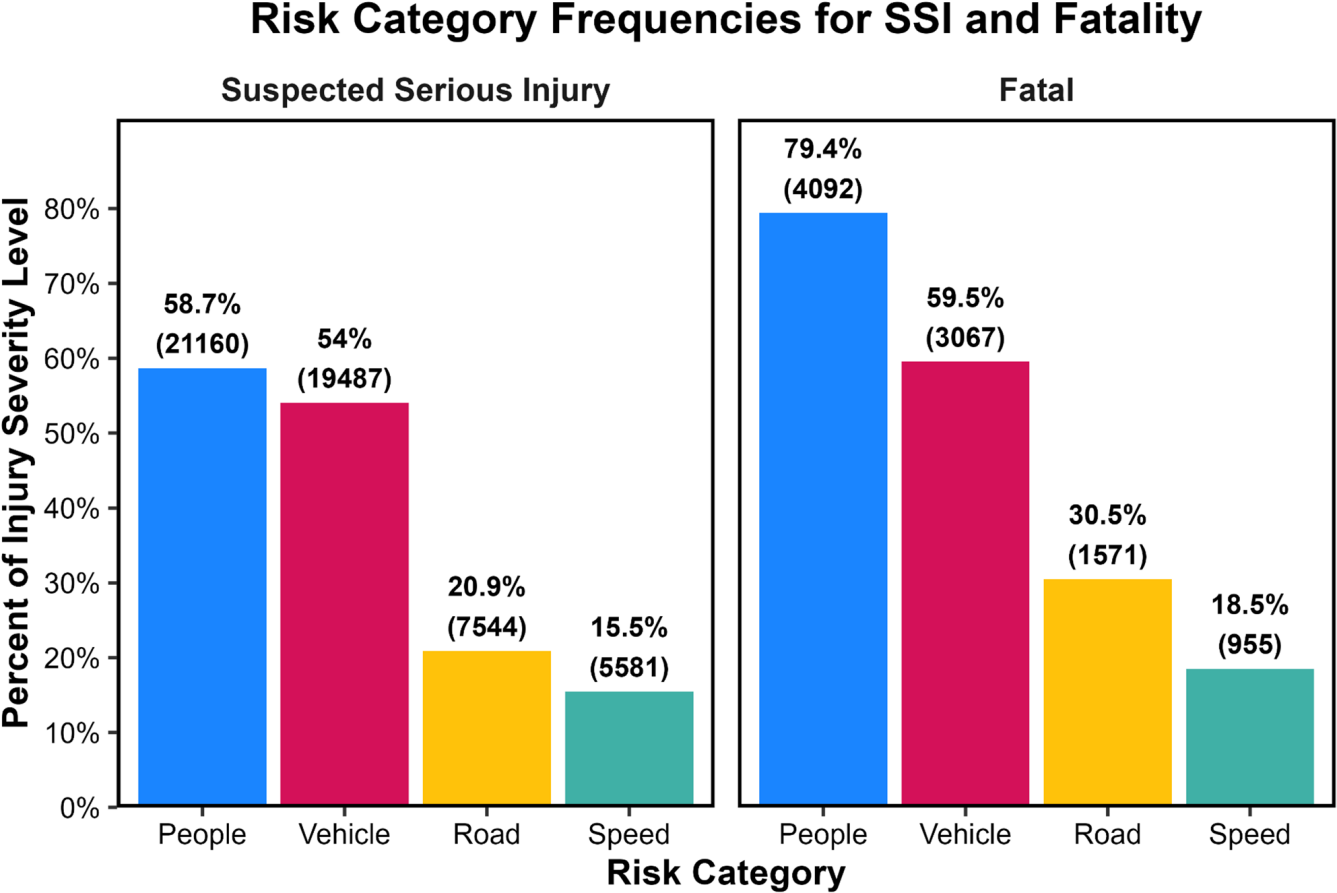
Relative frequency of SSIs (left graph) and fatalities (right graph) associated with each risk category (People, Vehicle, Road, Speed), shown as the percentage (bar height) of all SSIs (left graph) or fatalities (right graph) with raw counts in parentheses

**Fig. 2:**
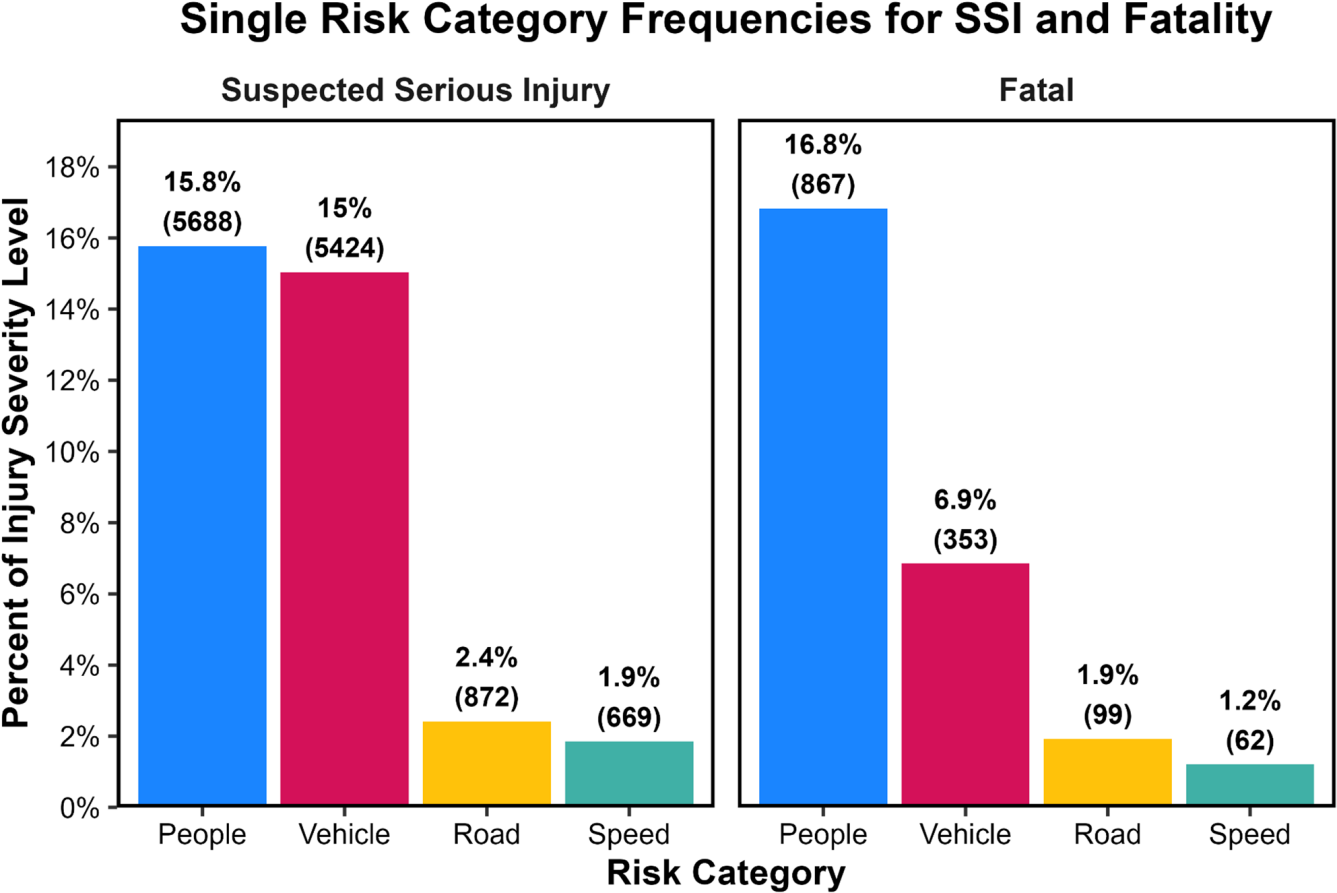
Relative frequency of SSIs (left graph) and fatalities (right graph) associated *only* with a single risk category, shown as the percentage of all SSIs (left graph) or all fatalities (right graph) with raw counts in parentheses

**Fig. 3:**
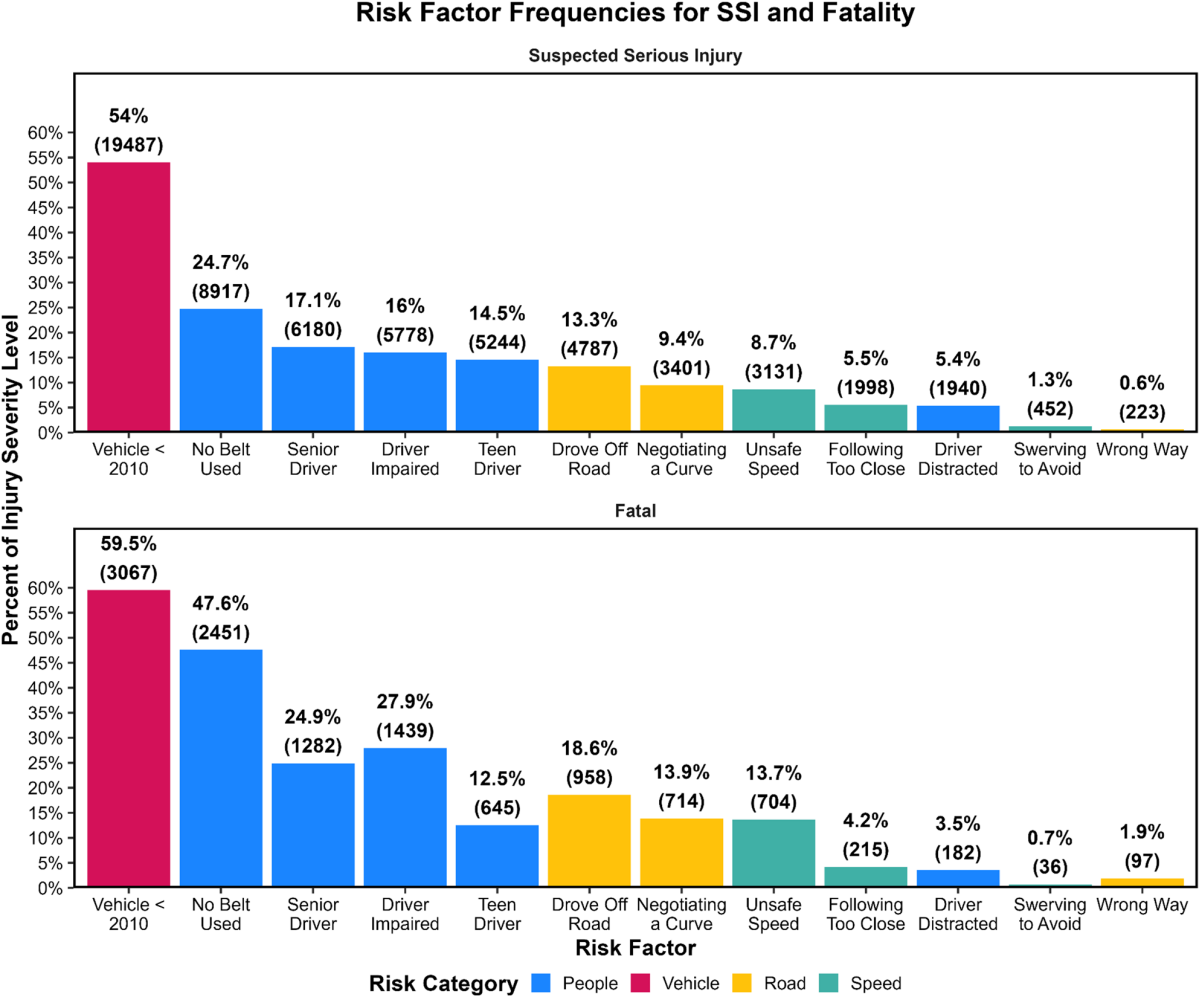
Relative frequency of SSIs (top graph) and fatalities (bottom graph) associated with each individual risk factor, shown as the percentage of all SSIs (top graph) or all fatalities (bottom graph) with raw counts in parentheses

**Fig. 4:**
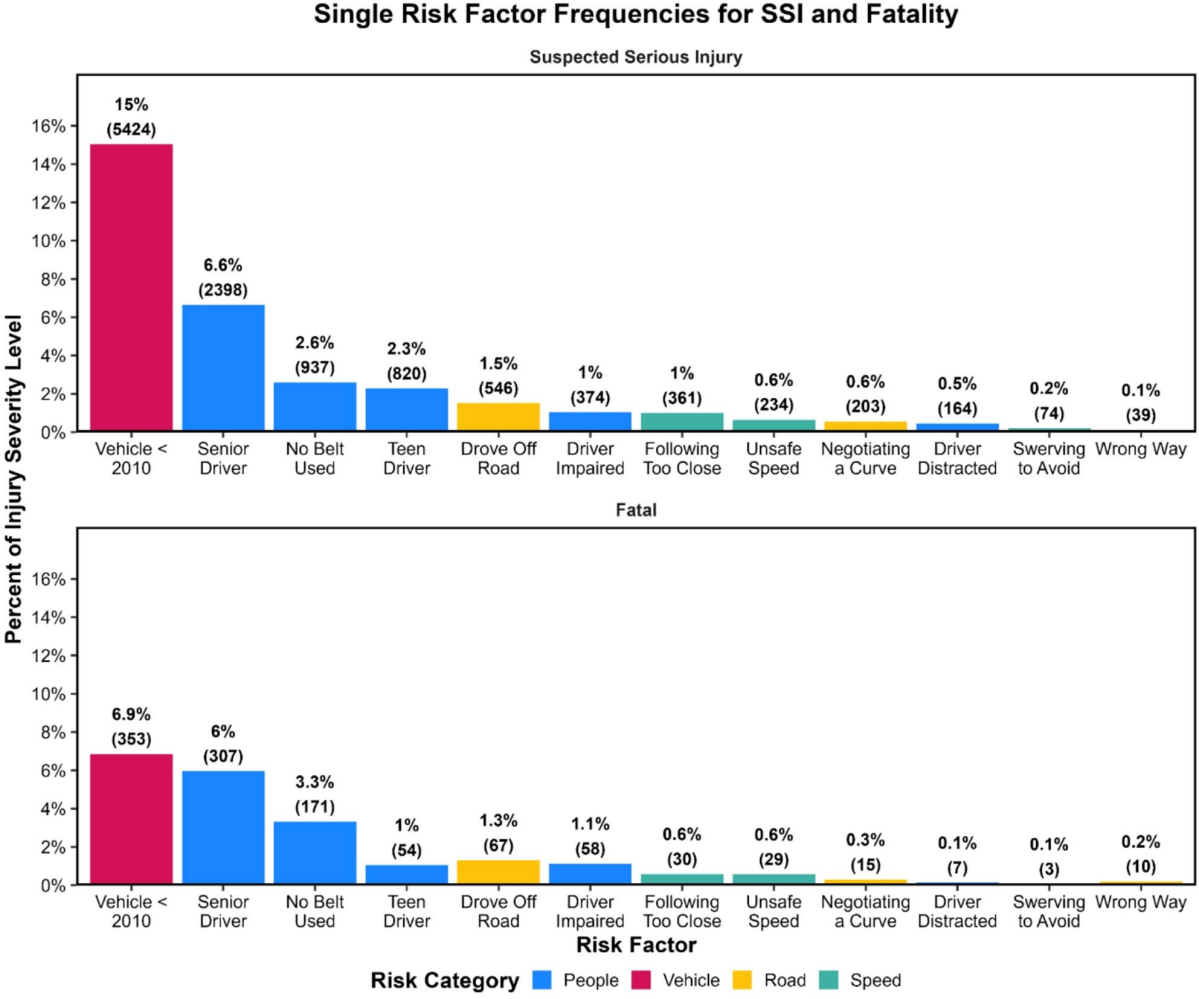
Relative frequency of SSIs (top graph) and fatalities (bottom graph) associated only with a single risk factor, shown as the percentage of all SSIs (top graph) or all fatalities (bottom graph) with raw counts in parentheses

**Fig. 5:**
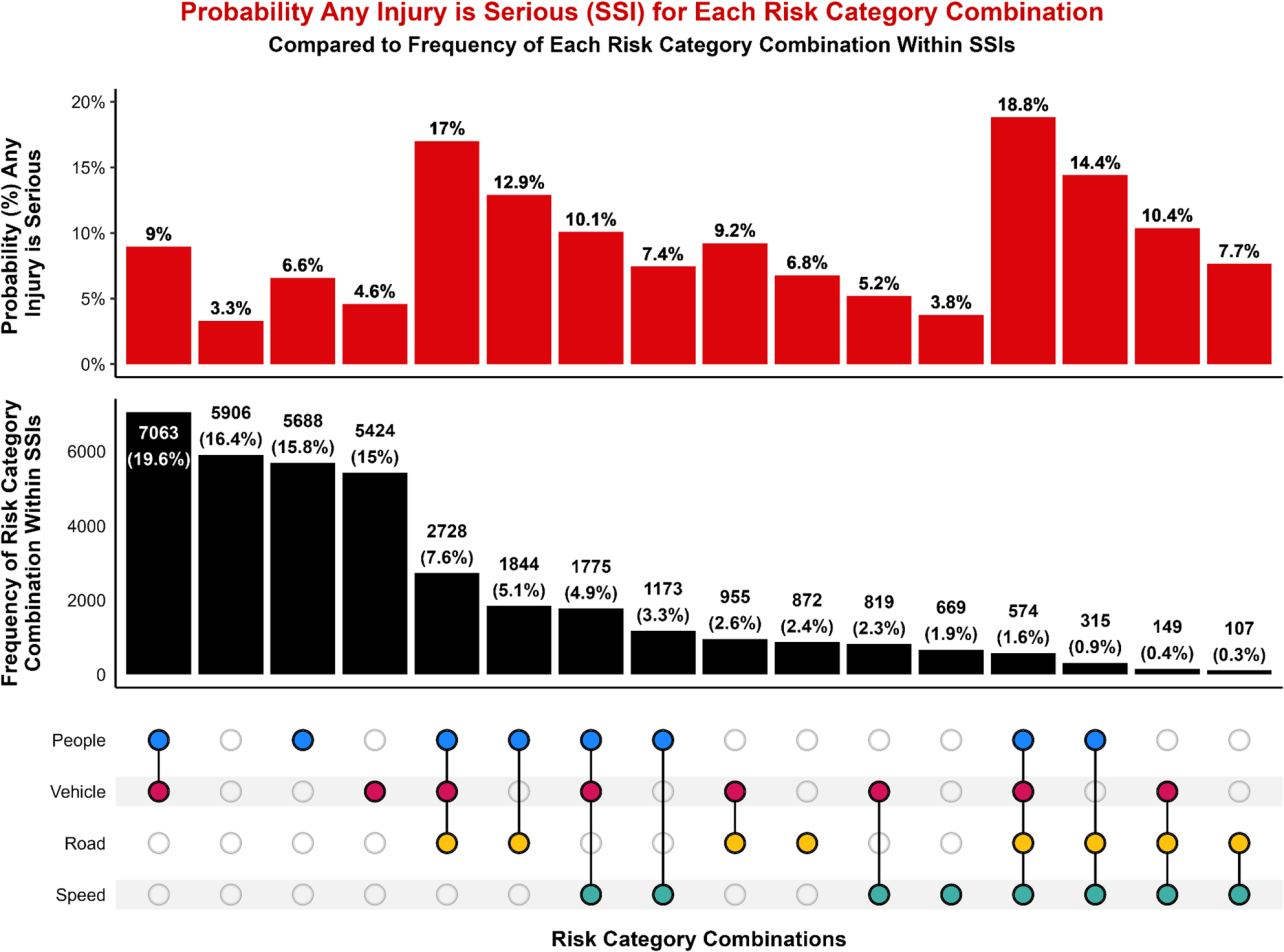
UpSet plot of risk categories (colored dots) and their possible combinations (black lines). Red bars show the predicted probability that any person-level injury is an SSI, given the associated combination of risk categories. Black bars show the frequency of SSIs associated with each risk category combination, quantified as a count and as a percentage of all SSIs. The column with blank dots represents SSIs with no associated risk categories

**Fig. 6:**
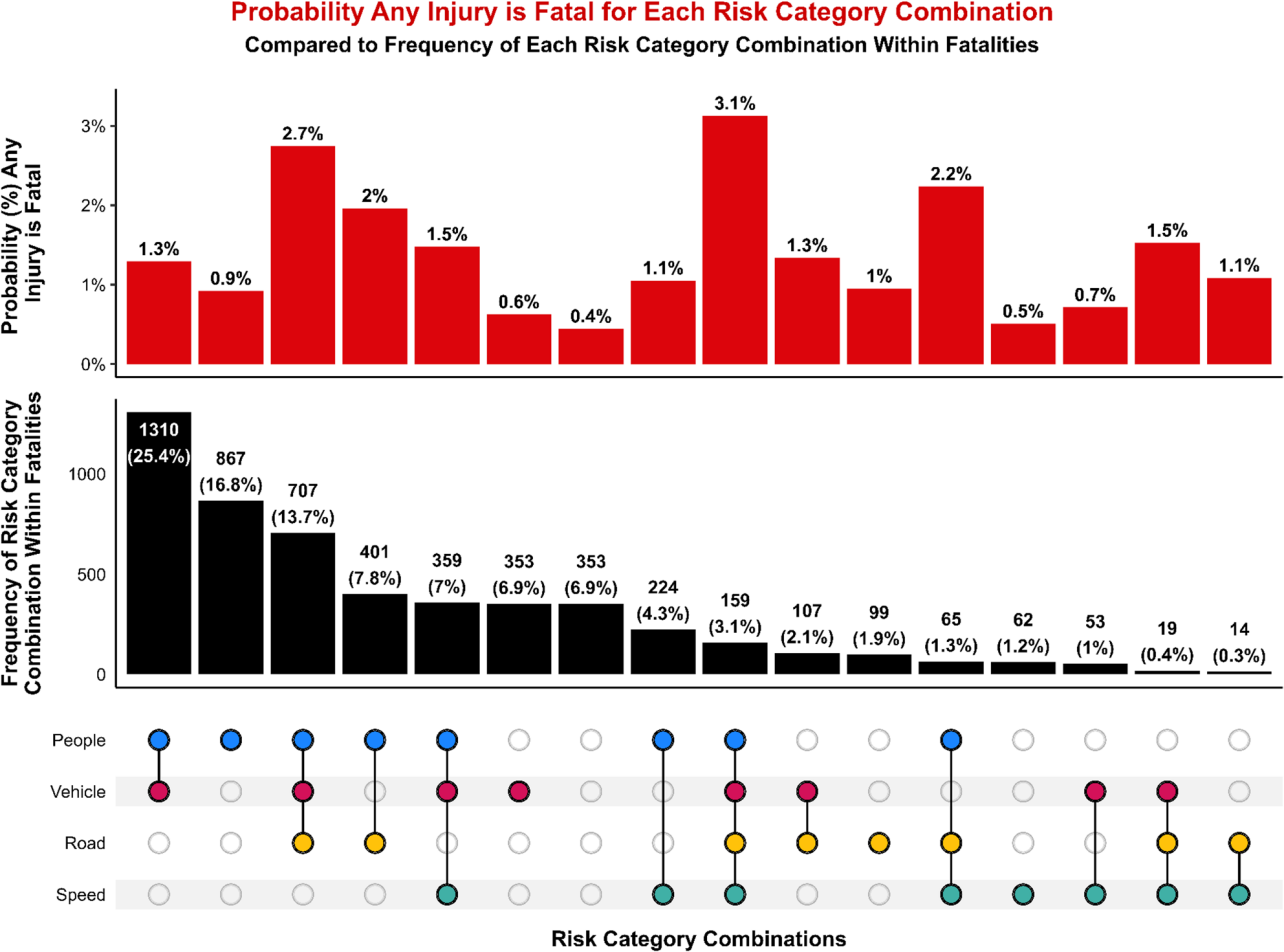
UpSet plot of risk categories (colored dots) and their possible combinations (black lines). Red bars show the predicted probability that any person-level injury is fatal, given the associated combination of risk categories. Black bars show the frequency of fatalities associated with each risk category combination, quantified as a count and as a percentage of all fatalities. The column with blank dots represents fatalities with no associated risk categories

**Fig. 7:**
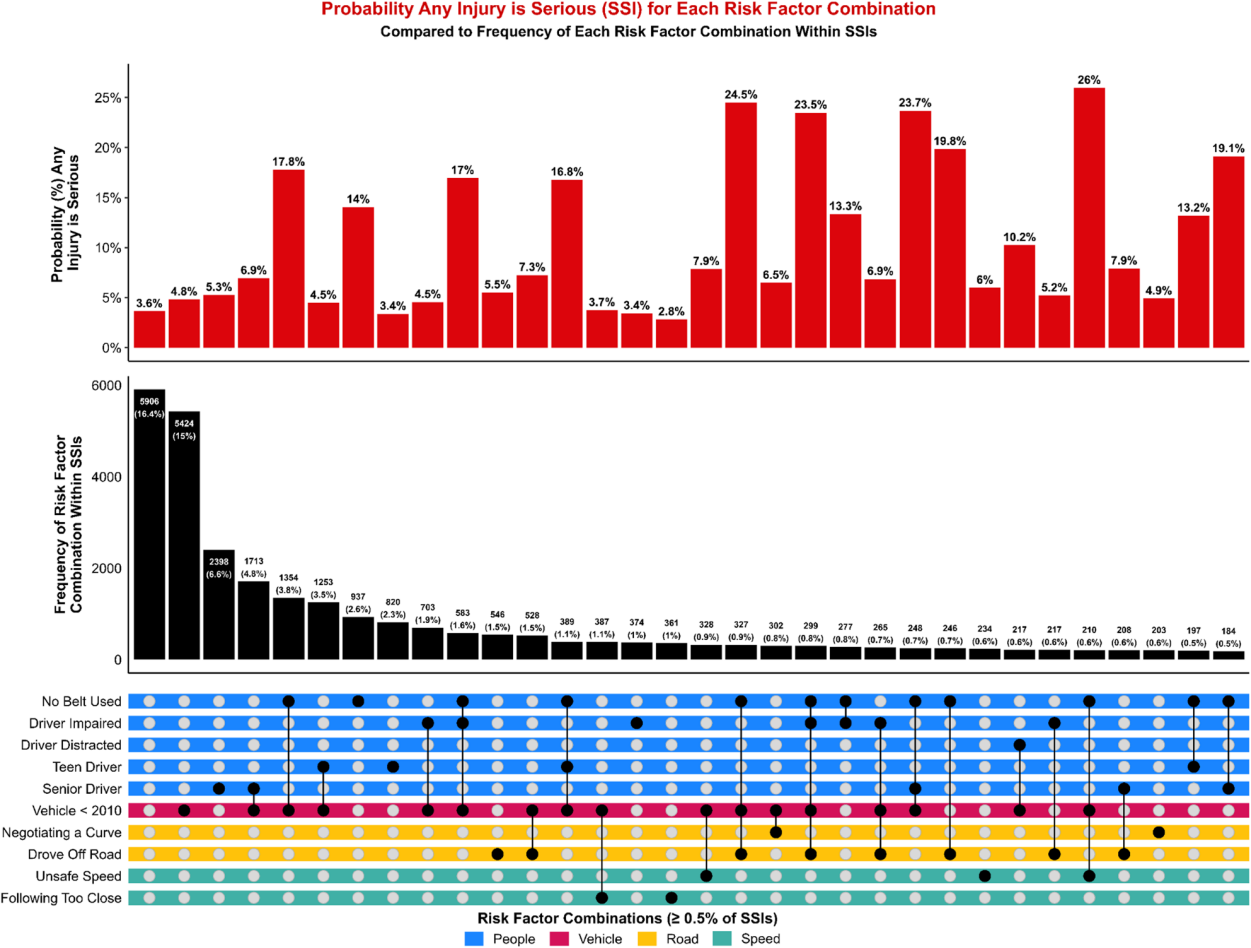
UpSet plot of individual risk factors (dots) and their combinations (lines), color-coded by risk category. Displayed risk factor combinations are associated with at least 0.5% of all SSIs. Red bars show the predicted probability that any person-level injury is an SSI, given the associated combination of risk factors. Black bars show the frequency of SSIs associated with each risk factor combination, quantified as a count and as a percentage of all SSIs. The column with blank dots represents SSIs with no associated risk factors

**Fig. 8:**
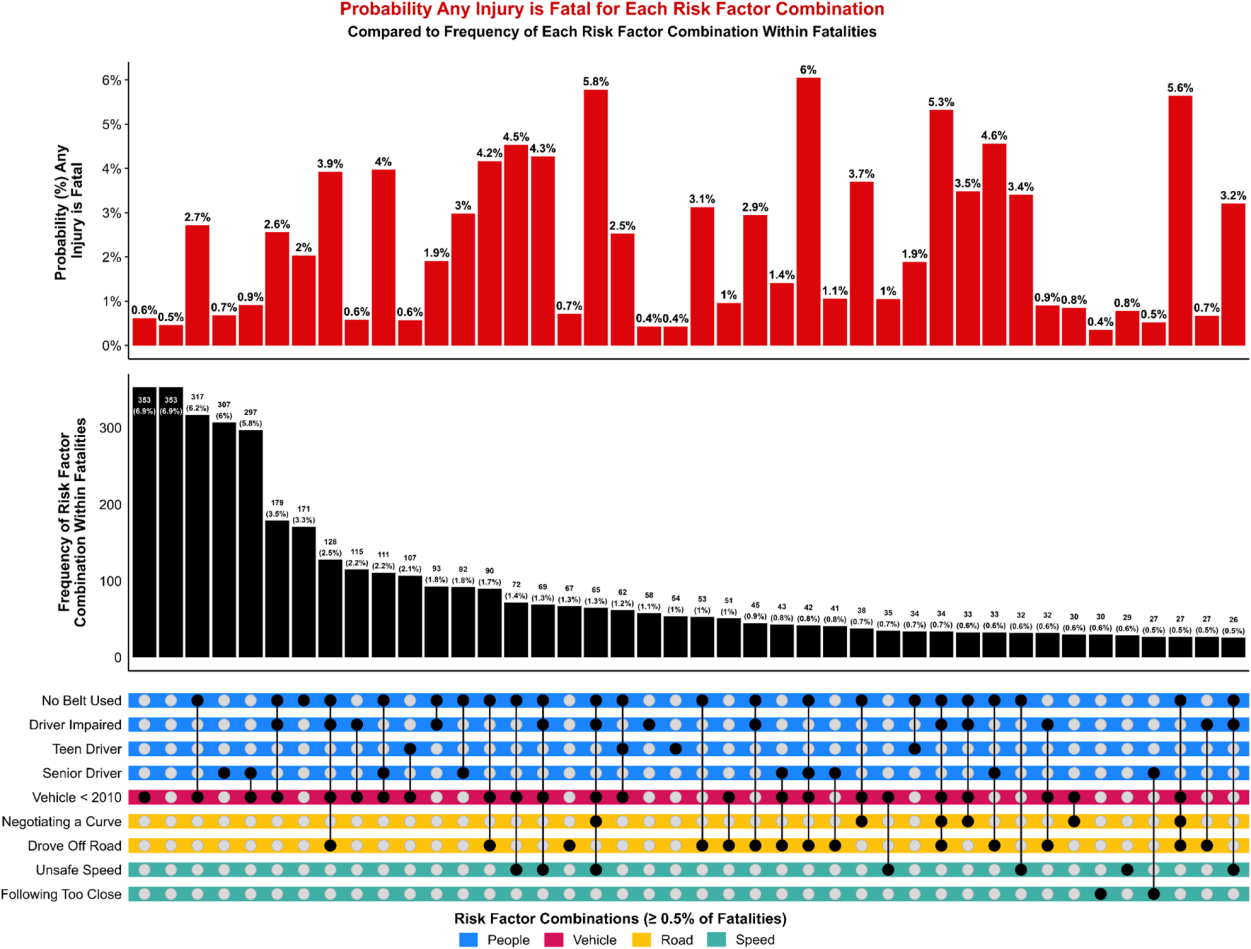
UpSet plot of individual risk factors (dots) and their combinations (lines), color-coded by risk category. Displayed risk factor combinations are associated with at least 0.5% of all fatalities. Red bars show the predicted probability that any person-level injury is fatal, given the associated combination of risk factors. Black bars show the frequency of fatalities associated with each risk factor combination, quantified as a count and as a percentage of all fatalities. The column with blank dots represents fatalities with no associated risk factors

This analysis examined the distribution of Safe System risk factors (Figs. 1, 2, 3 and 4), the frequency of risk factor combinations in individual cases of suspected serious injury and fatality, and their influence on the probability that any injury is serious or fatal (Figs. 5, 6, 7 and 8) across a statewide dataset of vehicle occupants injured in Ohio crashes from 2017 to 2023. The findings illustrate how individual conditions contribute to severity and, more importantly, how combinations of people-, vehicle-, road-, and speed-related factors shape vehicle occupant injury outcomes when they converge within the transportation system.

### Relative frequency of risk categories

Among occupants with *suspected serious injuries*, people-related factors were the most frequently documented, appearing in 58.7% of cases (Fig. 1). Vehicle-related factors were nearly as common, occurring in 54.0% of SSIs, while road-related factors and speed-related factors appeared in 20.9% and 15.5% of cases, respectively. *Fatalities* demonstrated a similar but more pronounced pattern: people-related factors were present in 79.4% of fatal crashes, vehicle-related factors in 59.5%, road-related factors in 30.5%, and speed-related factors in 18.5% (Fig. 1). These distributions indicate that most severe and fatal injuries occur in the presence of multiple system factors rather than single, isolated conditions.

When examining cases involving only one risk category, the proportions were substantially smaller (Fig. 2). For SSIs, injuries associated exclusively with people-related factors comprised 15.8% of cases, while vehicle-only injuries accounted for 15.0% (Fig. 2). Road-only and speed-only conditions were comparatively rare (Fig. 2). Fatal injuries followed a similar pattern, although people-only crashes represented a slightly larger share at 16.8% (Fig. 2). These patterns reinforce that serious and fatal outcomes usually arise when several system elements are involved simultaneously, not when a single factor is acting alone.

### Relative frequency of risk factors

Several individual factors were particularly common across injury outcomes. Older vehicle age (Vehicle Year < 2010) appeared most frequently, contributing to 54.0% of SSIs and 59.5% of fatalities (Fig. 3). Among people-related factors, lack of belt use was present in nearly one quarter of SSIs and almost half of all fatalities (Fig. 3). Driver impairment appeared in 16.0% of SSIs and 27.9% of fatalities (Fig. 3), while teen drivers and senior drivers together accounted for substantial proportions of both outcomes. These factors often co-occurred with one another or with roadway features, intensifying their combined impact on injury severity.

When restricted to cases involving only one factor, older vehicles again represented the largest share of both SSIs and fatalities (Fig. 4), emphasizing the widespread influence of fleet age. Isolated behavioral risks, such as impairment or belt nonuse, were more common among fatalities than among SSIs, whereas SSIs are more frequently involved isolated non-behavioral contributors such as vehicle age or roadway conditions.

### Combinations of risk categories

The co-occurrence of Safe System domains demonstrated clear differences between the frequency of risk factors and the predicted severity of injury when those risk factors combine. The most common combinations among SSIs involved risk factors from both the People and Vehicle categories, followed by cases involving only people-related or vehicle-related factors (Fig. 5). While these combinations had a high frequency in the SSI dataset, they did not correspond to the highest predicted probability of a severe injury. Instead, the highest predicted probability that any injury would be severe occurred when conditions from all four domains—People, Vehicle, Road, and Speed—were present simultaneously. Although rare (1.6% of all SSIs), this full-domain combination produced a probability of 18.8% that any injury in these circumstances would be severe, substantially higher than any single-domain or two-domain combination.

Fatalities exhibited a similar pattern (Fig. 6). People and Vehicle was the most frequently observed combination and accounted for 25.4% of fatal crashes, but this configuration was not associated with the highest predicted probability of fatality for any risk factor combination. The most severe outcomes were predicted when people-, vehicle-, road-, and speed-related factors co-occurred, yielding a predicted probability of 3.1% that any injury with this combination would be fatal. The next highest predicted probability of fatality for any injury (2.7%) was associated with the combination of people-, vehicle-, and road-related factors. These results demonstrate that the presence of multiple system-level vulnerabilities, rather than the presence of a single dominant factor, most strongly predicts fatal outcomes.

### Combinations of risk factors

When analysis shifted from categories to specific risk factors, distinct patterns emerged regarding the combinations most strongly associated with severe outcomes. Although older vehicle age remained the most common individual factor overall, the highest predicted probabilities of more severe outcomes for any injury were associated with combinations that layered behavioral risks onto vehicle and roadway vulnerabilities. The combination of no belt use, older vehicle age, and unsafe speed produced a predicted probability of 26.0% of an SSI outcome for any injury, despite representing less than 1% of observed SSI cases (Fig. 7). This pattern may reflect that crashes involving this combination of high-risk factors more commonly result in fatal injury rather than survival with serious injury; however, when these conditions did occur among SSIs, they were associated with a substantially elevated probability of serious injury. Similarly, combinations involving driver impairment, no belt use, older vehicles, and roadway departure produced probabilities of an SSI outcome for any injury exceeding 20%. Even in the absence of additional risk factors, belt nonuse alone was associated with a markedly elevated probability of 14.0% of an SSI outcome for any injury.

Fatality patterns reflected a similar dynamic (Fig. 8). While older vehicles accounted for the largest share of fatal cases overall, the most severe predicted outcomes involved multiple layered risks. The combination of no belt use, senior drivers, vehicle age < 2010, and roadway departure produced the highest predicted probability of a fatal outcome for any injury at 6.0%. These combinations were relatively infrequent but produced disproportionately severe harm when they occurred, illustrating how high-risk system configurations can yield extreme outcomes even in small portions of the crash population.

## Discussion

This study demonstrates that severe and fatal motor vehicle crash outcomes are strongly shaped by the convergence of conditions across the Safe System domains of People, Vehicle, Road, and Speed. While individual factors, such as restraint nonuse, driver impairment, older vehicle age, and roadway departure, were each meaningfully associated with injury severity, the most consequential risk emerged when multiple system vulnerabilities overlapped. These findings reinforce a core Safe System principle: severe harm is not an inevitable consequence of human error but arises when errors occur in environments lacking sufficient layers of protection.

The study period (2017–2023) spans substantial disruptions in travel behavior associated with the COVID-19 pandemic. This period was selected to maximize statistical power while maintaining consistency in crash reporting definitions, variable availability, and coding practices across years. These years represent the most recent complete data available at the time of analysis and capture pre-pandemic, pandemic, and post-pandemic travel periods. Prior research has documented significant temporal instability in crash frequency and severity during this period, including reductions in overall traffic volumes but increases in speeding and fatal crash risk [31–36]. Similar to those studies, the present analysis reflects elevated severity risk associated with unsafe speeds, even as exposure patterns changed. Because this study focuses on overall associations between key variables and crash outcomes, the data were pooled across years to increase sample size and stability of estimates; however, the inclusion of multiple travel periods helps account for potential temporal variation in crash patterns.

The dominant presence of people- and vehicle-related factors across both suspected serious injuries and fatalities highlights persistent behavioral and technological gaps within the transportation system. Older vehicles, identified in more than half of all severe injuries and fatalities, represent a substantial crashworthiness disparity within the statewide fleet. This finding aligns with existing literature showing that newer vehicles equipped with advanced safety features (e.g., electronic stability control, advanced airbags, and driver assistance technologies) are associated with reduced injury severity [37, 38]. Similarly, overrepresentation of belt nonuse and driver impairment among fatalities is consistent with longstanding evidence linking these behaviors to increased crash severity [39]. However, the modeling results extend prior work by demonstrating that the impact of these behavioral risks is not independent; rather their effect is amplified when combined with vehicle and roadway vulnerabilities. The highest predicted probability of an SSI for any injury occurred when no belt use, older vehicle age, and unsafe speeds converged, and the highest probability of a fatal outcome for any injury was observed when older vehicles, senior drivers, restraint nonuse, and roadway departure occurred simultaneously. These combinations illustrate how risk is compounded when behavioral, vehicular, and environmental weaknesses align, an interaction effect that has been suggested but less frequently quantified in prior studies [40–42].

A key finding of this study is the clear divergence between frequency and predicted severity. Many combinations of factors were common but associated with comparatively lower predicted severity, while other combinations, particularly those involving multiple domains, were rare yet highly injurious. Traditional frequency-based approaches to crash analysis may therefore overlook the system conditions that pose the greatest threat to life. By estimating conditional probabilities of the severity level of any injury, this study builds on emerging methodological approaches that prioritize risk-based and outcome-focused metrics over simple crash counts [43]. These patterns highlight the importance of shifting from analyses that focus solely on individual risk factors toward frameworks that recognize how multiple system elements interact to produce severe outcomes.

The results of this study also offer meaningful insights for transportation planning, enforcement, engineering, and safety program development. The finding that older vehicles are both widespread and strongly associated with severe outcomes suggests value in fleet modernization strategies [44], including programs that support equitable access to safer vehicles [45]. This is consistent with prior research highlighting disparities in access to newer, safer vehicles across socioeconomic groups [46]. Elevated predicted severity for roadway departures reinforces the need for context-sensitive roadway design [47–49], including high-visibility markings, rumble strips, and speed management [50] in areas where vehicles are likely to leave the travel lane. The strong influence of belt nonuse and impairment, particularly when combined with other factors, emphasizes the continued need for targeted behavioral interventions and enforcement strategies. These interventions may be most effective when focused on high-risk contexts and populations rather than broad, undifferentiated approaches.

More broadly, these findings support a systemic approach to crash reduction by identifying conditions in which protective layers fail simultaneously. This aligns with a growing body of literature advocating for integrated, multi-domain safety strategies over single-factor interventions [43]. A Safe System–aligned strategy requires coordinated efforts across agencies and disciplines, focusing on environments where behavioral risks intersect with technological and roadway vulnerabilities. Interventions that address only a single emphasis area are unlikely to achieve the reductions in severe harm that can be reached when countermeasures are aligned across multiple domains. By explicitly quantifying how these domains interact, this study contributes to the evidence base supporting more holistic, system-level approaches to transportation safety.

### Limitations

Several limitations should be considered when interpreting the findings of this study. As with all police-reported crash data, the OH-1 system is subject to misclassification and inconsistent reporting, particularly for behavioral variables such as distraction, impairment, and restraint use. These factors are known to be underreported or documented differently across jurisdictions, which may lead to conservative estimates of their association with injury severity. The analysis also excluded pedestrians, bicyclists, and motorcyclists due to their distinct exposure profiles and injury mechanisms, meaning the results apply only to occupants of enclosed vehicles.

In addition, injury severity in the OH-1 system is determined by the responding law enforcement officer at the scene using observable indicators and standardized KABCO-aligned definitions. Classifications such as suspected serious injury (A) are based on visible or reported conditions (e.g., suspected fractures, severe lacerations, unconsciousness) rather than definitive clinical diagnosis. Consequently, the assigned severity level reflects an on-scene assessment rather than a medically confirmed outcome derived from hospital records or trauma registries. Prior research has documented discrepancies between police-reported injury classifications and medical diagnoses, particularly in differentiating suspected serious from minor injuries. Misclassification may therefore occur in both directions, potentially attenuating or inflating associations between risk factors and severity. However, because the OH-1 criteria are applied consistently statewide and reflect real-time field assessment, they remain appropriate for population-level injury surveillance and comparative risk modeling, even if they do not precisely correspond to clinical injury scales such as AIS or ISS.

The analysis also excluded pedestrians, bicyclists, and motorcyclists due to their distinct exposure profiles and injury mechanisms, meaning the results apply only to occupants of enclosed vehicles.

Some variables required consolidation across multiple OH-1 fields, particularly those within the People and Road domains. This approach was necessary for statewide standardization but may introduce heterogeneity within each risk factor. Similarly, the designation of Vehicle Year < 2010 was derived statistically as a proxy for diminished crashworthiness and the absence of modern safety technologies; however, it does not directly measure specific vehicle safety features or maintenance status. Other potentially important influences, such as socioeconomic factors, travel exposure, seat position, and detailed roadway geometry—were not available within the statewide dataset and therefore could not be incorporated into the modeling framework.

The predicted probabilities reported in this study reflect conditional associations rather than causal relationships. Co-occurrence of factors indicates that multiple system elements were present during the same crash, but it does not imply statistical interaction or causal influence unless explicitly modeled. The goal of this analysis was to characterize real-world constellations of vulnerability, not to determine causal pathways. Finally, the findings are specific to Ohio’s fleet composition, roadway network, enforcement environment, and reporting practices. States with newer vehicle fleets, different roadway designs, or alternate crash-reporting standards may exhibit different patterns of risk. Nonetheless, the breadth of the dataset and the consistency of observed patterns across seven years suggest that the overarching findings are robust and likely generalizable to similar jurisdictions.

Lastly, future research should examine temporal instability directly by stratifying models by pre-pandemic and pandemic periods or by applying time-varying or interaction modeling approaches to assess whether predictors of KA crashes shifted during and after COVID-19.

## Conclusion

This study illustrates that serious and fatal motor vehicle crash outcomes are driven by interactions among risk factors across the Safe System domains of People, Vehicle, Road, and Speed. While individual factors such as belt nonuse, impairment, older vehicle age, or roadway departure are important predictors, the most severe outcomes occurred when these factors converged. This finding reflects the core Safe System principle that human errors need not result in fatal consequences when the transportation system includes multiple layers of protection.

By quantifying conditional probabilities for specific combinations of risk factors, the study provides a practical, data-driven framework for identifying system vulnerabilities and guiding integrated countermeasures. Strategies that simultaneously address behavioral risks, modernize the vehicle fleet, enhance roadway design, and manage travel speeds are likely to produce greater reductions in SSIs and fatalities than any single-domain approach. The overrepresentation of older vehicles and the elevated severity associated with behavioral factors in high-speed or roadway departure contexts underscore the need for equitable vehicle-safety initiatives, targeted infrastructure improvements, and context-sensitive speed management. Together, these insights support a shift toward systemic risk reduction as the foundation for future transportation safety efforts.

The findings of this study provide actionable guidance for transportation agencies and safety practitioners. By identifying the specific combinations of risk factors associated with the highest predicted probabilities of SSI and fatality, the analysis enables agencies to target interventions where they are most likely to yield substantial reductions in severe outcomes. Corridors where older vehicles, roadway departures, and unsafe speeds intersect may warrant prioritized engineering improvements, such as enhanced markings, rumble strips, or speed-calming measures. Behavioral insights, particularly the elevated risk associated with belt nonuse and impairment, can support focused enforcement and public education campaigns tailored to high-risk populations or contexts.

The modeling framework is readily scalable because it relies on standardized crash-report variables and transparent statistical methods. Other jurisdictions can adopt this approach to characterize their own system-level vulnerabilities and design tailored Safe System–aligned strategies. By integrating behavioral, vehicle, roadway, and speed-related considerations, agencies can move beyond siloed emphasis areas and toward coordinated, multi-domain safety programs capable of reducing serious injuries and fatalities systemwide.

## Data Availability

The data supporting the findings of this study are publicly available through the Dryad Digital Repository. De-identified crash-level, unit-level, and person-level data used in this analysis were obtained from the Ohio Department of Public Safety and curated into a publicly accessible dataset: Harden, Angela; Cole, Mary (2026). Modeling Injury Severity Among Motor Vehicle Occupants Using a Safe System–Aligned, Population-Based Framework: Evidence from Ohio Crash Data (2017–2023). figshare. Dataset. https:/doi.org/10.6084/m9.figshare.31164676.v1.
